# Sleeve gastrectomy with anti-reflux procedures

**DOI:** 10.1590/S1679-45082014AO2885

**Published:** 2014

**Authors:** Sergio Santoro, Arnaldo Lacombe, Caio Gustavo Gaspar de Aquino, Carlos Eduardo Malzoni

**Affiliations:** 1 Hospital Israelita Albert Einstein, São Paulo, SP, Brazil.; 2 Faculdade de Ciências Médicas da Santa Casa de São Paulo, São Paulo, SP, Brazil.

**Keywords:** Obesity/surgery, Gastrectomy/methods, Gastroesophageal reflux

## Abstract

**Objective:**

Sleeve gastrectomy is the fastest growing surgical procedure to treat obesity in the world but it may cause or worsen gastroesophageal reflux disease. This article originally aimed to describe the addition of anti-reflux procedures (removal of periesophageal fats pads, hiatoplasty, a small plication and fixation of the gastric remnant in position) to the usual sleeve gastrectomy and to report early and late results.

**Methods:**

Eighty-eight obese patients that also presented symptoms of gastroesophageal reflux disease were submitted to sleeve gastrectomy with anti-reflux procedures. Fifty of them were also submitted to a transit bipartition. The weight loss of these patients was compared to consecutive 360 patients previously submitted to the usual sleeve gastrectomy and to 1,140 submitted to sleeve gastrectomy + transit bipartition. Gastroesophageal reflux disease symptoms were specifically inquired in all anti-reflux sleeve gastrectomy patients and compared to the results of the same questionnaire applied to 50 sleeve gastrectomy patients and 60 sleeve gastrectomy + transit bipartition patients that also presented preoperative symptoms of gastroesophageal reflux disease.

**Results:**

In terms of weight loss, excess of body mass index loss percentage after anti-reflux sleeve gastrectomy is not inferior to the usual sleeve gastrectomy and anti-reflux sleeve gastrectomy + transit bipartition is not inferior to sleeve gastrectomy + transit bipartition. Anti-reflux sleeve gastrectomy did not add morbidity but significantly diminished gastroesophageal reflux disease symptoms and the use of proton pump inhibitors to treat this condition.

**Conclusion:**

The addition of anti-reflux procedures, such as hiatoplasty and cardioplication, to the usual sleeve gastrectomy did not add morbidity neither worsened the weight loss but significantly reduced the occurrence of gastroesophageal reflux disease symptoms as well as the use of proton pump inhibitors.

## INTRODUCTION

Both gastroesophageal reflux disease (GERD) and obesity present a major increase in incidence in the world. They are often associated, especially because obesity increases the intra-abdominal pressure, generating the forces necessary to cause the reflux.^(1,2)^

Sleeve gastrectomy (SG) was seen just as a part of the biliopancreatic bypass with duodenal switch (BPD-DS). In 2003, it was first suggested^(3)^ that the SG (without intestinal interventions) could be an early treatment for obesity, by interrupting its progression, in cases in which clinical treatment could not stop it, possibly avoiding more aggressive procedures in the future. Also for the first time, SG was seen as a metabolic and adaptive procedure^(3,4)^ rather than a restrictive one that poses obstacles to food ingestion, like narrow anastomoses or bands.

In the same period, some high-risk patients, waiting for a BDP-DS were submitted to the SG first, leaving the BPD for later.^(5,6)^ Unexpected good results were observed.^(7)^ Soon, SG was being considered as an isolated procedure to treat obesity^(8-10)^ due to the nice association of physical and neuroendocrine modifications. Because SG may produce excellent results achieving very high quality of life with smaller changes in the general structure of the gastrointestinal tract, it has become very popular,^(11-13)^ with an increasing number of surgeries worldwide.

However, there are some reports that SG may cause or worsen GERD, causing the appearance of hiatal hernias^(14)^ and physical and functional damage to the lower esophageal sphincter (LES),^(15)^ although there is some controversy.^(16)^

## OBJECTIVE

To describe an innovative association of usual anti-reflux procedures, consisting of the removal periesophageal fat pads, hiatoplasty, and small plication, applied immediately before a sleeve gastrectomy. Later, there was the fixation of the remnant gastric pouch in position. This association was called “anti-reflux sleeve gastrectomy”. Secondly, to report its impact on symptoms of reflux and weight loss, in a retrospective comparison to the sleeve gastrectomy without these anti-reflux procedures.

## METHODS

### Patients

Eighty-eight patients with body mass index (BMI) at the moment of the surgery varying from 33.4 to 51kg/m^2^, with a primary complaint of obesity but also presenting gastroesophageal reflux were submitted to anti-reflux SG (ARSG). Fifty of them were also submitted to a transit bipartition (ARSG + BT). BT is a partial biliopancreatic bypass in which the duodenum is not divided, preserving its transit and function, therefore diminishing the malabsorption associated to complete biliopancreatic bypasses, but maintaining an early nutrient stimulus to the distal gut. BT is used as a mean to potentiate the results of a SG.^(17,18)^

Preoperative exams included upper gastrointestinal endoscopy and esophageal manometry. Some were also submitted to upper gastroesophageal radiography using oral barium as a contrast (upper gastrointestinal series) especially those whose endoscopic exams pointed the existence of hiatal hernias. Those presenting esophageal motility problems (other than those related to GERD itself), symptoms of dysphagia or Barret esophagus were not included.

Post-operatively, since most did not present symptoms, just upper gastrointestinal series were provided for all. More invasive exams, such as endoscopy and manometry, were not generally applied.

Register of weight loss (in terms of percentage of excessive BMI loss – EBMIL %) was collected using a software, especially developed for collecting data after bariatric surgeries. Results were compared to 360 patients that received SG and 1,140 patients with SG + TB from our data bank that did not receive any procedure to treat GERD.

All patients submitted to ARSG (38) and ARSG + TB (50) were localized and specifically inquired about their current symptoms of GERD and the use of proton pump inhibitors (PPIs). Symptoms were classified, in relation to the pre-operative status as worse, unaltered, better or asymptomatic. The use of PPIs was described as none, sporadic (not continuous) or continuous. The occurrence of dysphagia was also actively inquired.

To obtain comparable groups that were not submitted to anti-reflux procedures, we also specifically inquired 50 patients submitted to SG alone and 60 submitted to SG + BT that presented some preoperative complain of GERD. Additionally, 50 patients submitted to SG alone that did not have any GERD symptoms pre-operatively were inquired to check if SG could induce GERD in previously non-symptomatic patients. They were operated on between 2006 and 2011, and the questionnaire included symptoms of GERD and the use of PPIs. Control groups included patients between 19 and 64 years old and BMI between 35 and 48kg/m^2^.

### Procedure

Under laparoscopy, first, the fat pads that cover the His angle and surround the esophageal-gastric transition were excised and removed for a better exposition of this point. Then, the omental bursa was opened and the greater omentum was divided utilizing a 5-mm sealer and divider device (Ligasure^®^ or Ultracision^®^). Dissection started along the gastric greater curvature at a middle point, going up to the angle of His, releasing completely the gastric fundus, until the left arm of the diaphragmatic esophageal hiatus was well exposed. Then, from its right side, the esophagus was isolated and surrounded by a narrow Penrose drain to help with its traction, mobilization and exposure, bringing the stomach completely to the abdominal cavity, if there was a hiatal hernia. The right arm of the hiatus was also dissected and exposed. Fat pads that coul existed around the distal esophagus (and may be very voluminous) were removed. A 32F boogie was passed to the stomach.

A hiatoplasty was performed as usual, with two or more stitches of non-absorbable material. The cardioplication was then constructed. Since the gastric fundus should be left out of the plication, this procedure was put in a little lower position, very close to the Penrose, marking the gastroesophageal transition. Indeed it did not involve the fundus properly but the region of the cardia. Therefore, we called it a cardioplication to differ from a usual fundoplication.

**Figure 1:**
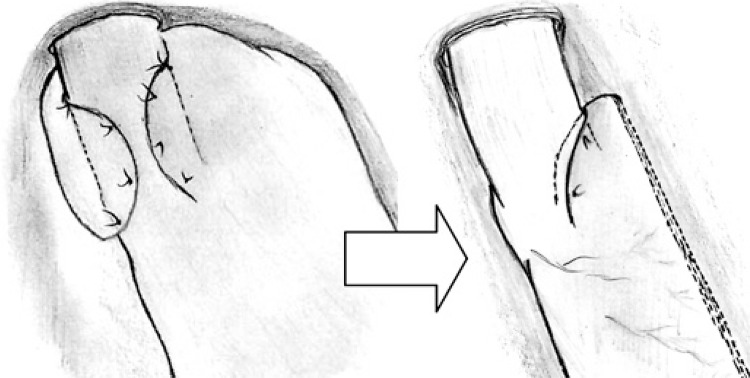
The traditional partial fundoplication (left) used in the surgical treatment of gastroesophageal reflux disease and the cardioplication (right): a lower position, a shorter plication and just 180o embracement spare most of the gastric fundus allowing a sleeve gastrectomy

In a fundoplication, the esophagus was embraced around 3 or 4cm above the transition, utilizing much of the gastric fundus. Here, the transition was dissected a little lower, in the small curvature, and the plication embraced the esophagogastric transition and a small part of the distal esophagus. Also, the cardioplication embraced mainly the left aspect of the esophagus, around 180^o^. Typically, four stiches are used, one posteriorly, one anteriorly and two in the same level between them (forming a half circle). This cardioplication was smaller than the traditional partial fundoplication, but it aimed to keep and protect the angle of His (and the sling fibers that it contains) from stapling (Figure 1), while it still allowed the resection of the fundus.

The lower part of the greater curvature was then dissected until a point located 2 to 3cm from the pylorus, to allow the stapling to begin 4cm from the pylorus. A 32-F Fouchet’s tube was pushed until the antrum to guarantee that the gastric tube was at least 3cm wide (always wider than the esophagus). SG was performed with a 60-mm articulating laparoscopic linear stapler, not very tight against the Fouchet tube, to prevent the stretching of the stomach wall. The articulation of the stapler allowed all the stapling process to be done from the umbilical port, but sometimes the last firings were done from the left subcostal port, if it was easier. The cardioplication was obviously spared. Therefore, it was fundamental that this plication was built before the stapling, not after. Special care was taken not to create a narrowing below the plication to avoid the formation of a sand-clock shaped stomach.

To prevent bleeding, a non-absorbable invaginating seromuscular running suture was made to completely cover the stapling line. Recently, with better staples (Tri-Staples^®^),^(19)^ we leaved some without this covering suture. Fifty of them were additionally submitted to a transit bipartition as previously described,^(16,17)^ to enhance weight loss and remission of comorbidities in the heavier patients and in the more affected by metabolic syndrome.

The omentum was fixed with a few simple stitches to “the new greater curvature” to assure that the stomach stayed in the right position,^(20)^ without coiling.

A suction drain (Blake^®^ or similar) was used and it was exteriorized at the site of the left flank 5-mm trocar. The specimen was retrieved through the umbilicus. Skin incisions were closed with absorbable intradermic sutures and covered with glue (Dermabond^®^). Just at the umbilical site, the aponeurosis was sutured to avoid hernias.

Antibiotic and deep vein thrombosis prophylaxis was used in all patients. They received only intravenous fluids in the first 24 to 36 hours and then, they were instructed to take just liquids for 15 days. After, they were allowed to progressively start eating soft solids. PPIs were kept for 30 days in all patients, independently of symptoms.

This work was submitted and approved by the Research Ethics Committee (protocol 506.070). It did not require a special consent term, other than the usual applied to all surgical patients. It was conducted at the *Hospital Israelita Albert Einstein*, actively inquiring patients operated between 2004 and 2013.

### Statistical analysis

To compare the evolution of GERD symptoms, SG and SG + TB were analyzed together and compared to ARSG and ARSG + TB together. Patients that become worst and unaltered were grouped and compared to those that became better and asymptomatic. Comparison was obtained by using Pearson’s χ^2^ test for proportions. The utilized software was the R Core Team (Vienna, 2013).

To compare the average weight loss in each period separately, 3 and 6 months, 1, 2, 3, 4 and 5 years, means and 95% confidence interval were used.

## RESULTS

The patients for ARSG (38 patients) and ARSG + TB (50 patients) were submitted to surgery by all four authors, from the same group, from 2004 to 2012. Follow-up was 3 to 89 months (mean 22 months). Fifty-six were females, and 32 males. The mean preoperative BMI for ARSG was 38.7kg/m^2^ and for ARSG + TB, 41.8kg/m^2^, while the 360 patients with a SG had an average BMI of 39.2kg/m^2^, and 42.3kg/m^2^ for 1,140 patients submitted to ARSG + TB.

For 38 patients with ARSG, the mean EBMIL% reduction was 52%±4, 74%±5, 79%±7, 72%±8, 75%±7, 62%±8, and 60%±8, respectively at 3, 6 months, 1, 2, 3, 4 and 5 years, while for SG alone (360 patients), it was respectively 49%±3, 72%±5, 84%±6, 79%±7, 74%±7, 60%±7, and 57%±8.

**Figure 2:**
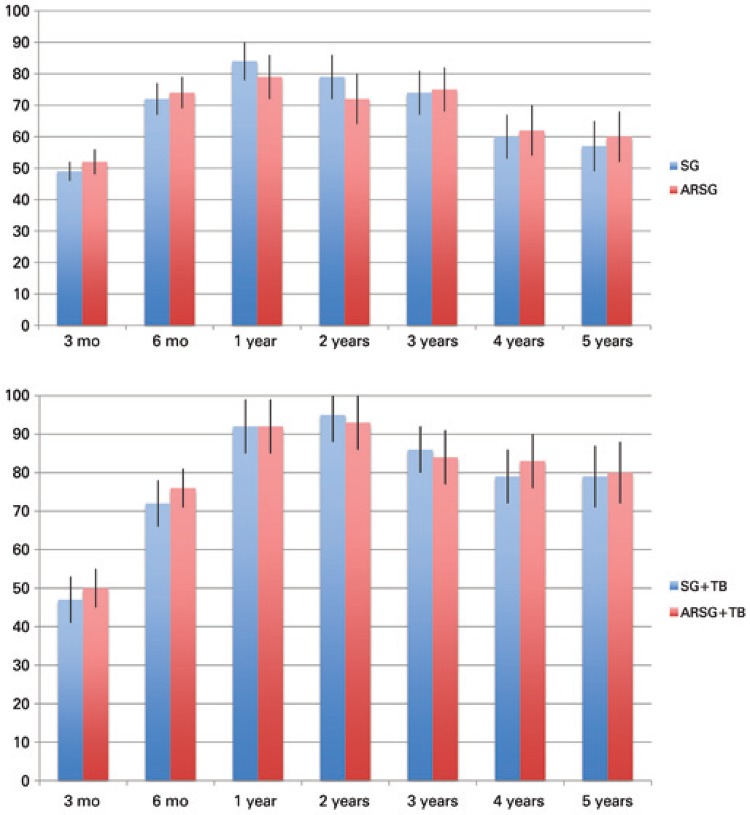
Left, a comparison of the percentage of excessive body mass index between the usual sleeve gastrectomy (blue columns) and the anti-reflux sleeve gastrectomy (red columns). In the graphic at the right, a comparison between percentage of excessive body mass index of sleeve gastrectomy + partial biliopancreatic bypass (blue columns) and anti-reflux sleeve gastrectomy + partial biliopancreatic bypass (red columns)

For ARSG + BT average EBMIL% reduction was 50%±5, 76%±5, 92%±7, 93%±7, 84%±7, 83%±7, and 80%±8 respectively at 3, 6 months, 1, 2, 3, 4 and 5 years, while for SG + BT without procedures to treat or prevent GERD the observed results are 47%±6, 72%±6, 92%±7, 95%±7, 86%±6, 79%±7, and 79%±8 (Figure 2).

Using the 95% interval confidence, statistics demonstrate that in all periods analyzed in terms of weight loss, ARSG + TB was not inferior to SG + TB alone.

Using the same statistic method, comparing SG alone with ARSG, the sample size was smaller (38 patients), and in two periods (1 and 2 years postoperatively) it was not possible to state that ARSG was not inferior to SG, but in all other periods (3 months, 6 months, 3, 4 and 5 years) the weight loss obtained with ARSG was not inferior to SG alone.

### Evolution of gastroesophageal reflux disease symptoms

The 50 patients submitted to SG alone that did not have GERD symptoms preoperatively, 19% started presenting some symptoms, especially in the first 3 months after the operation. They required the use of PPIs (sporadic or non-continuously 13%; continuously 6%). Therefore, SG could induce symptoms of GERD in those who previously did not have them.

Among patients who had the typical GERD symptoms (heartburn or reflux sensation) preoperatively, patients with SG and SG + BT with no procedures to treat or prevent GERD were analyzed together.

In relation to the complaints related to GERD previously to surgery, 15% worsened, 38% remained unaltered, 41% improved and just 6% referred complete relief of symptoms. Thus, SG could either improve or worsen the symptoms. This group of patients was already on PPIs preoperatively. After surgery in this group, just 12% no longer used PPIs; while 88% used them (19% continuously, and 78% sporadically).

**Figure 3:**
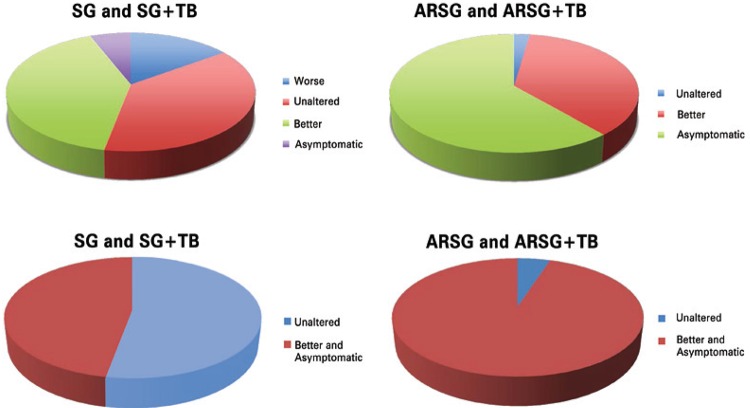
Comparison of evolution of GERD symptoms between usual SG or SG+TB and ARSG or ARSG+TB (above). Below a simplified analysis is shown where “worse and unaltered” are joined, as well as “better and asymptomatic”. Statistical difference is observed. Patients submitted to anti-reflux procedures had significantly less symptoms (P<0,001)

Now referring to the patients that received ARSG, in terms of GERD symptoms they were also analyzed together (ARSG and ARSG + TB) and all of them had symptoms before surgery. After ARSG, none answered that was worse, 2 (2.3%) were unaltered, 32 (36.4%) were better, and 54 (61.4%) were without symptoms. For statistical purposes worst and unaltered were grouped, as well as better and asymptomatic as shown in figure 3. Compared with the symptomatic patients who did not undergo hiatoplasty with cardioplication, these results are significantly better (p<0.001).

This was also shown when the use of PPIs was analyzed. From 100% use of PPIs, either continuously or sporadically in this group, the use fell to 36.3% (2.3% continuously and 34% sporadically); 63.7% do not need PPIs anymore (p<0.01).

Persistent dysphagia to solids occurred only in one patient after ARSG but no reintervention was performed. Surgical complications were rare: one case of post-operative bleeding that demanded transfusion, but not reoperation in ARSG, and one case of intestinal sub-occlusion in ARSG + TB, that did not demanded reoperation either.

Preoperative manometry in ARSG and ARSG + TB demonstrated an abnormally low mean respiratory pressure in the LES, varying from 4 to 15mmHg (8.6±3.2mmHg). It was rarely used in the postoperative period due to its low acceptance by asymptomatic patients. Therefore, these data were not used for a comparison.

**Figure 4:**
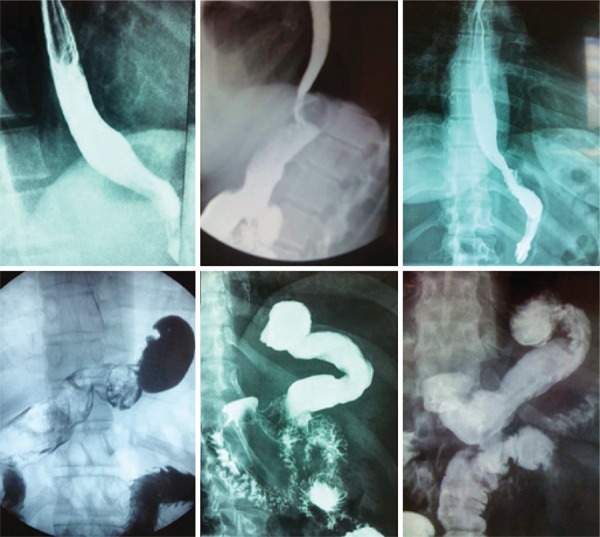
Upper GI radiographies with barium swallow after ARSG. Three top images show patients in upright position. Three images at the bottom show patients in a Trendelemburg position (it is observed that contrast accumulates at the top of the stomach) and Valsava maneuver (the stomach is compressed against the diaphragm). No esophageal reflux was seen even under adverse conditions. The inferior right image is ARSG+TB: observe the latero-lateral gastroileal anastomosis in the antrum

Upper gastroesophageal radiography using oral barium as a contrast (upper gastrointestinal series) was the most used as means to objectively evaluate surgical results. All hiatal hernias (21 out of 88 patients) were no longer detected. Fifty patients have been submitted to upper gastrointestinal series (Figure 2): 44 normal exams, just observing the SG and the plication (Figure 4) and 6 exams pointed some mild reflux; 5 also showed some esophageal tertiary waves. Valsava maneuver and Trendelenburg position are part of the protocol in upper gastrointestinal series and some patients, even in this adverse condition presented no reflux during the exam (Figure 4; observe that in Trendelenburg position and during Valsava maneuver, stomachs appear to be larger than in usual upstanding position and the contrast medium is most in the upper part of the stomach). The 38 patients without an objective postoperative test were completely asymptomatic.

## DISCUSSION

SG is becoming increasingly more frequent and important as a digestive surgical procedure. A detailed international survey about bariatric procedures pointed that between 2008 and 2011, SG was the only with an increase in the absolute number of procedures, and not by little: it increased from 18,098 to 94,689, a increase of 523%, while all the others diminished in absolute numbers.^(13)^

SG has been proven to be safe and effective, causing significant weight loss and improving an expressive number of metabolic conditions, including diabetes.

However, SG has its flaws. The weakest point in this procedure is the fact that, in some patients, it may cause or worsen GERD. The second major flaw is that it may not be enough for all patients as a treatment of severe obesity and severe metabolic syndrome. Some patients may need more (interventions that involves the gut additionally).

In relation to GERD symptoms, some patients improve from its symptoms after SG while others get worse, as mentioned in the literature^(14-16,21)^ and confirmed here. The implications of a SG in GERD are multiple and very complex.^(21)^

There are arguments in favor of an improvement of GERD after SG. Indeed, patients loose weight what helps improving GERD. But there are some other theoretical explanations for this improvement that can occur before weight loss.^(16)^ SG removes most oxyntic cells, which probably significantly reduces acid production (although it might be obvious, it was never properly proved). Additionally, SG theoretically reduces the tension on the gastric walls below the cardia at the same interior pressure, as it reduces the radius of the gastric fundus. The law of LaPlace was invoked for this explanation:^(16,20)^ the larger the vessel radius, the larger the wall tension required to withstand a given internal pressure. The tension in cardia walls is the force that opposes to the action of the LES. Some authors have well documented an improvement in LES pressure,^(16)^ prior to the weight loss, that could reflect this physical hypothesis.

But, in opposite direction, SG elevates intragastric pressure as it reduces gastric compliance.^(22)^ If the intragastric pressure is much increased, even with a small radius, wall tension may increase and reflux may appear, or get worse.

In SG, the faster gastric emptying is demonstrated at the beginning of meals,^(23)^ what is positive in preventing and treating GER, but at a certain point, gastric emptying is reduced (mostly because of the action of gut hormones, like GLP-1)^(24)^ and intragastric pressure obviously raises. It is a complex scenario of pros and cons.

There is another crucial point in this discussion involving SG and GERD. Beginning in 1980, there was a revolution in the understanding of GERD. Before 1980, reflux was considered a result of a persistently weak LES. Dent et al. discovered that most GER events, both in normal subjects and GERD patients, were a product of brief relaxations of the LES. These were later called transient LES relaxations (TLESRs).^(25,26)^

TLESRs are the most important mechanism for the occurrence of GER. TLESRs are relaxations that are not triggered proximally as those that follow swallows. TLESRs are triggered distally, by gastric distension, they last longer and they occur simultaneously to a relaxation of diaphragmatic crura. They occur in normal people and they are responsible for belching, a physiological event.^(26)^ The lean GERD patients present more frequent TLESRs as well as morbidly obese patients do.^(27)^

TLESRs occur periodically and are triggered by neurally controlled myoeletric events generated in the gastric fundus and cardia.^(26)^ Therefore, it is possible that the resection of the gastric fundus removes the sources of TLESRs.

Comparing patients that were submitted to usual Nissen fundoplications, TLESRs (triggered by gastric fundus distension by air) were significantly higher in patients having their short gastric vessels intact, than those that had them divided.^(28)^ This suggests that simply dividing vessels (and neural connections) cause a diminution in TLESRs. It is reasonable to accept that a SG may cause a major diminution in TLESRs, but this still has to be demonstrated in both, the typical SG and in the ARSG.

If some more of the fundus is maintained after a SG, its distension could start triggering frequent TLESRs. Indeed, in the SG group we observed two patients that developed extremely frequent belching right after SG, what took months to disappear. These were probably caused by undesirable more frequent TLESRs.

By this rationale, the best SG would be the one in which the stapling line is very close to the esophageal gastric junction. This is exactly what Petersen et al.^(16)^ observed and suggested, in the same article, which states that SG enhances LES pressure.

However, getting close to the esophageal gastric junction might be dangerous, not just because the risk of fistula, but also because of the danger of damaging the sling fibers of the LES. Braghetto et al.^(15)^ in an excellent article clearly showed that it is possible to damage these fibers, reducing LES basal pressure and causing GER.

In this complex scenario, to obtain the best SG is not easy because it is not wise to staple too close to the esophageal gastric junction, neither too far.

After many decades of successful anti-reflux surgery, we have data about the effect of fundoplications on the TLESRs. Both partial^(29)^ and complete (Nissen)^(30^^)^ fundoplications reduce the occurrence of TLESRs. Anti-reflux surgery is an effective treatment against GERD. It prevents GER efficiently and complete fundoplication sometimes impedes belching too.^(30)^

Frequently, surgeons associate the efficacy of a fundoplication with the amount of wrapping around the esophagus, meaning that a 360° wrapping would be superior to a 270°, which in turn would better than a 180°. There is no objective support to this idea. Indeed, in a recent broad meta-analysis^(31)^ the opposite was shown and the 180° fundoplication presented superior overall results.

ARSG, as proposed here, removes fat pads around the esophagus, corrects hiatal hernias, protects the esophageal gastric junction from being cut, therefore it protects the sling fibers and it quite maintains the angle of His. The plication still aims at maintaining the small diameter of the “new gastric tube”, providing less wall tension (less distension, as a consequence) of the remnant cardia. Additionally, it creates traction at the LES level.

Objectively, ARSG caused a very expressive reduction in the symptoms of GERD and in the necessity of PPIs when compared to SG (both with and without TB). Although we do not have enough data regarding the changes in pressure of the LES neither in the frequency of TLESRs, ARSG was effective in reducing GERD symptoms. Obviously, to know exactly how this little plication affects LES pressure and TLESR frequency would enrich this discussion and specific studies are needed, but on the other hand there is scientific support to spare asymptomatic patients from invasive tests as symptom follow-up evaluation is also adequate after fundoplications, and that routine physiologic testing is not necessary to asymptomatic patients^(32)^ (especially because they do not want to repeat it).

ARSG + BT was applied to heavier patients, those with more intense metabolic syndrome or those with severe limitations to exercise, in whom ideal weight loss is less probable and more important. The weight loss (in terms of EBMIL%) was better than ARSG alone and not worse than in SG + BT without anti-reflux procedures, in all periods examined, demonstrating that the cardioplication did not impair weight loss. TB creates a wide gastroileal anastomosis that may prevent the elevation of intragastric pressure after SG and as a consequence, the results relative to GERD could be even better, but this was not proven yet.

Tai et al.^(14)^ well pointed that after a SG and the expected weight loss, hiatal frequently hernias appear. It is reasonable, since usually there is a lot of fat around the gastroesophageal transition that may diminish, leaving a very loose hiatus. ARSG removes the fat pads and corrects eventual hiatal hernias, assuring that the EG junction is in the abdomen and, by closing the crura, possibly attenuating the intensity of diaphragmatic relaxation that occurs simultaneously to TLESRs, as it is also supposed to happen in the hiatoplasty of usual fundoplications.

By fixing the stomach in position after SG,^(20)^ stomach coiling may be prevented. This may contribute to a lower gradient pressure to obtain the gastric emptying. This fact, however, although intuitive, was never objectively proven. To facilitate the gastric emptying is recognized as a part of GERD treatment.

This article originally described that some usual and simple procedures in the surgical treatment of GERD can be applied to a SG. The attached video shows them (http://learning.einstein.br/ao2885). The observation of the results in this group is stimulating. However, the study is retrospective and non-randomized and precise objective measures were not demonstrated. It demands additional studies.

## CONCLUSION

The addition of antireflux procedures (hiatoplasty, fat pads removal, fixing the stomach in the right position and the cardioplication) to the usual sleeve gastrectomy in this group did not add morbidity neither worsened the weight loss but significantly reduced the occurrence of gastroesophageal reflux symptoms as well as the use of proton pump inhibitors. Additional prospective and randomized studies are needed to further evaluate these technical modifications.
