# Supplementary material for: Sleeve gastrectomy with anti-reflux procedures
Source: Einstein (Sao Paulo). 2014 Jul-Sep;12(3):287–94. doi: 10.1590/S1679-45082014AO2885 (PMC4872937; doi:10.1590/S1679-45082014AO2885)
Supplement: Translation

# Translation: Gastrectomia vertical com medidas antirrefluxo

**Authors:** Sergio Santoro, Arnaldo Lacombe, Caio Gustavo Gaspar de Aquino, Carlos Eduardo Malzoni

**Affiliations:** 1 Hospital Israelita Albert Einstein, São Paulo, SP, Brasil.; 2 Faculdade de Ciências Médicas da Santa Casa de São Paulo, São Paulo, SP, Brasil.

**Keywords:** Obesidade/cirurgia, Gastrectomia/métodos, Refluxo gastroesofágico

## Abstract

**Objetivo:**

A gastrectomia vertical é o procedimento cirúrgico para tratamento da obesidade que mais cresce em indicações. No entanto, esse procedimento pode causar ou agravar a doença do refluxo gastresofágico. Este artigo buscou descrever originalmente a adição de procedimentos antirrefluxo (remoção de coxins gordurosos do hiato, hiatoplastia, pequena plicatura e fixação do remanescente na posição anatômica), além de relatar seus resultados precoces e tardios.

**Métodos:**

Foram submetidos à gastrectomia vertical com medidas antirrefluxo 88 oito pacientes obesos com sintomas de doença do refluxo gastresofágico. Dentre esses pacientes, 50 foram submetidos também à bipartição do trânsito. A perda de peso destes foi comparada àquela ocorrida em 360 pacientes consecutivos submetidos à gastrectomia vertical usual e à de 1.140 pacientes submetidos à gastrectomia vertical + bipartição do trânsito. Os sintomas do refluxo gastresofágico foram investigados por meio de questionário em todos os submetidos à gastrectomia vertical com medidas antirrefluxo e comparados com os resultados obtidos em 50 pacientes submetidos à gastrectomia vertical usual e a 60 submetidos à gastrectomia vertical + bipartição do trânsito, também com sintomas prévios de doença do refluxo gastresofágico.

**Resultados:**

O percentual de perda do excesso de índice de massa corporal após gastrectomia vertical antirefluxo não foi inferior a gastrectomia vertical usual. Além disso, a gastrectomia vertical antirefluxo + bipartição do trânsito não foi inferior a gastrectomia vertical + bipartição do trânsito. Não houve aumento da morbidade na gastrectomia vertical antirefluxo, porém notou-se redução significativa dos sintomas da doença e do uso de inibidores de bomba prótons para tratar a condição.

**Conclusão:**

A adição de procedimentos antirrefluxo, como a hiatoplastia e a cardioplicatura, à gastrectomia vertical usual não acrescentou morbidade nem piorou a perda de peso obtida, mas diminuiu de modo significativo a ocorrência de sintomas da doença do refluxo no pós-operatório, assim como a utilização de inibidores de bomba de prótons.

## INTRODUÇÃO

Tanto a doença do refluxo gastresofágico (DRGE) como a obesidade apresentam importante aumento em incidência no mundo. Geralmente, elas estão associadas, pois a obesidade aumenta a pressão intra-abdominal, que gera as forças necessárias para causar o refluxo.^(1,2)^

A gastrectomia vertical (GV) era vista apenas como parte da derivação biliopancreática com *switch *duodenal (DBP-SD). Em 2003, sugeriu-se pela primeira vez^(3)^ que a GV (sem intervenção duodenal) poderia ser a conduta inicial para obesidade, interrompendo sua progressão em casos em que o tratamento clínico não obtivesse êxito e, assim, evitando procedimentos mais agressivos no futuro. Igualmente, pela primeira vez, a GV foi vista como um procedimento metabólico e adaptativo,^(3,4)^ e não restritivo.

No mesmo período, alguns pacientes de alto risco que aguardavam DBP-SD foram submetidos primeiramente à GV, postergando a DBP.^(5,6)^ Bons resultados inesperados foram observados.^(7)^ Logo, a GV foi considerada um procedimento isolado para tratar obesidade^(8-10)^ graças à boa associação entre modificações físicas e neuroendócrinas. Como a GV pode produzir excelentes resultados e proporcionar ótima qualidade de vida com mudanças menores na estrutura geral do trato digestório, ela está se tornando muito popular,^(11-13)^ com números crescentes no mundo todo.

Todavia, há relatos de que GV pode causar ou piorar a DRGE por aparecimento de hérnias hiatais,^(14)^ e de lesão física e funcional ao esfíncter esofágico inferior (EEI).^(15)^ Contudo, esse tema ainda é controverso.^(16)^

## OBJETIVO

Descrever uma associação inovadora de procedimentos antirrefluxo comuns, que consistiu em remoção de coxins adiposos periesofágicos, hiatoplastia e pequena plicatura, aplicados imediatamente antes da gastrectomia vertical. Posteriormente, houve fixação do reservatório gástrico remanescente na posição anatômica. Tal associação foi chamada “gastrectomia vertical antirrefluxo”. Em seguida, é relatado seu impacto nos sintomas de refluxo e perda de peso, em uma comparação retrospectiva às gastrectomias verticais sem esses procedimentos antirrefluxo.

## MÉTODOS

### Pacientes

Foram submetidos a GV antirrefluxo (GVAR) 88 pacientes com índice de massa corporal (IMC) no momento da cirurgia variando de 33,4 a 51kg/m^2^ e com queixa principal de obesidade, mas também apresentando refluxo gastresofágico. Destes, 50 pacientes foram também submetidos à bipartição do trânsito (GVAR + BT). BT consiste em uma derivação biliopancreática parcial na qual o duodeno não é seccionado, preservando seu trânsito e sua função, e diminuindo a má absorção associada a derivações biliopancreáticas completas, mas mantendo um estímulo nutricional precoce ao intestino distal. BT é utilizada como forma de potencializar os resultados da GV.^(17,18)^

Exames pré-operatórios incluíram endoscopia digestiva alta e manometria esofágica. Alguns pacientes foram também submetidos à radiografia gastresofágica alta usando bário como contraste (série de trato digestivo alto), em especial aqueles cujos exames endoscópicos apontaram a existência de hérnia de hiato. Já os que apresentaram problemas de motilidade esofágica (diferentes daqueles relacionados diretamente à DRGE), sintomas de disfagia ou esôfago de Barret não foram incluídos.

No pós-operatório, como a maioria não apresentava sintomas, apenas imagens de trato digestivo superior foram realizadas. Os exames mais invasivos, como endoscopia e manometria, não foram realizados em todos os pacientes.

O registro de perda de peso (em termos de perda de excesso de IMC percentual − EBMIL%) foi feito com um *software *especialmente desenvolvido para coleta de dados pós-operatórios. Os resultados foram comparados a 360 pacientes que fizeram GV e a 1.140 GV + BT de nossa base de dados que não fizeram nenhum procedimento para tratar DRGE.

Todos os pacientes submetidos a GVAR (38) e à GVAR + BT (50) foram localizados e inquiridos especificamente sobre seus sintomas atuais de DRGE, bem como sobre uso de inibidores da bomba de prótons (IBPS). Os sintomas foram classificados em relação ao estado pré-operatório como pior, inalterado, melhor ou assintomático. O uso de IBPs foi descrito como nenhum, esporádico (não contínuo) ou contínuo. A ocorrência de disfagia também foi ativamente questionada.

Para obter grupos comparáveis que não tivessem sido submetidos a procedimentos antirrefluxo, também questionamos especificamente 50 pacientes que realizaram GV apenas, e 60 submetidos à GV + BT que apresentavam alguma queixa pré-operatória de DRGE. Além disso, 50 pacientes que realizaram apenas GV e que não tinham nenhum sintoma pré-operatório de DRGE foram questionados para checar se a GV poderia induzir DRGE em pacientes previamente assintomáticos. Eles foram operados entre 2006 e 2011, e o questionário incluiu sintomas de DRGE e uso de IBPs. Os grupos controle incluíram pacientes de 19 a 64 anos, e com IMC entre 35 e 48kg/m^2^.

### Procedimento

Sob laparoscopia, primeiramente os coxins adiposos que recobrem o ângulo de His e cercam a transição esofagogástrica foram excisados e removidos para melhor exposição desse ponto. A seguir, o epiplon foi aberto e o grande epiplon foi dividido, utilizando-se um dispositivo de 5mm para seccionar e selar (Ligasure^®^ ou Ultracision^®^). A dissecção começou ao longo da grande curvatura gástrica, em um ponto no meio, subindo em direção ao ângulo de His e liberando completamente o fundo gástrico, até que o braço esquerdo do hiato esofágico diafragmático estivesse bem exposto. Então, a partir do lado direito, o esôfago foi isolado e abraçado por um dreno de Penrose estreito, para auxiliar sua tração, mobilização e exposição, trazendo o estômago completamente para a cavidade abdominal, caso houvesse hérnia de hiato. O braço direito do hiato também foi dissecado e exposto. Coxins de gordura que pudessem existir em torno do esôfago distal (e ser bastante volumosos) foram removidos. Uma sonda 32F foi passada no estômago.

Uma hiatoplastia foi feita da forma usual, com dois ou mais pontos de fio de sutura não absorvível. A cardioplicatura foi, então, realizada. Pela necessidade de deixar o fundo gástrico fora da plicatura, esta foi colocada em posição um pouco mais baixa, bem próxima ao Penrose, que marcava a transição gastresofágica. Não envolveu o fundo propriamente, mas a região do cardia. Foi chamada de cardioplicatura para diferenciar da fundoplicatura convencional.

Em uma fundoplicatura, o esôfago foi abraçado cerca de 3 ou 4cm acima da transição, utilizando boa parte do fundo gástrico. Aqui, a transição foi dissecada um pouco mais abaixo, na pequena curvatura, e a plicatura abraçou a transição esofágica e uma pequena porção do esôfago distal. Também a cardioplicatura envolveu principalmente o lado esquerdo do esôfago, cerca de 180°. O modo convencional é usar quatro pontos de sutura - um posterior, um anterior e dois no mesmo nível entre eles (formando um círculo). Essa cardioplicatura é menor do que a fundoplicatura tradicional parcial, mas seu objetivo é manter e proteger o ângulo de His (e as fibras oblíquas que ele contém) dos grampos (Figura 1), enquanto permite ainda a ressecção adequada do fundo gástrico.

**Figura 1:** A fundoplicatura parcial tradicional (esquerda) utilizada no tratamento cirúrgico da doença do refluxo gastresofágico e a cardioplicatura (direita): uma posição mais baixa, uma plicatura menor e o envolvimento de 180o poupam a maior parte do fundo gástrico e permitem a gastrectomia vertical

A porção mais inferior da pequena curvatura foi, assim, dissecada até um ponto localizado de 2 a 3cm do piloro, para permitir começar o grampeamento a 4cm do piloro. Uma sonda de Fouchet 32F foi passada até o antro, para garantir que o tubo gástrico tivesse pelo menos 3cm de largura (sempre mais amplo que o esôfago). Uma GV foi feita com grampeador linear laparoscópico articulado de 60mm, não muito justo contra o Fouchet, para evitar esticar a parede do estômago. A articulação do grampeador permitiu que todo o processo de grampeamento fosse feito a partir da porta umbilical, mas, por vezes, as últimas cargas eram feitas a partir da porta subcostal esquerda, se assim fosse mais fácil. A cardioplicatura foi obviamente preservada. Desse modo, foi fundamental que essa plicatura fosse feita antes do grampeamento, e não depois. Houve cuidado especial para não criar um estreitamento abaixo da plicatura, para evitar a formação de estômago em ampulheta.

Para evitar sangramento, foi feita uma sutura contínua não absorvível seromuscular para cobrir completamente a linha de grampos. Recentemente, com grampos melhores (Tri-Staples^®^),^(19)^ foram deixados alguns sem essa sutura de cobertura. Cinquenta pacientes foram também submetidos à bipartição do trânsito como previamente descrito,^(16,17)^ para aumentar a perda de peso e a remissão de comorbidades nos pacientes mais pesados e naqueles mais afetados por síndrome metabólica.

O epiplon foi fixado com poucos pontos simples à “nova grande curvatura”, para garantir que o estômago ficasse na posição correta,^(20)^ sem torcer.

Um dreno de sucção (Blake^®^ ou similar) foi utilizado e exteriorizado no local do trocarte de 5mm do flanco esquerdo. A peça foi retirada através do umbigo. Incisões de pele foram fechadas com suturas intradérmicas absorvíveis e cobertas com cola (Dermabond^®^). Apenas na porta de entrada do umbigo, a aponeurose foi suturada para evitar hérnias.

Foram usados antibióticos e profilaxia para trombose venosa profunda em todos os pacientes. Receberam apenas líquidos por via intravenosa nas primeiras 24 a 36 horas e, então, foram instruídos a ingerir apenas líquidos por 15 dias. Após, os pacientes puderam progressivamente começar a ingerir alimentos pastosos. Os IBPs foram mantidos por 30 dias em todos os pacientes, independentemente dos sintomas.

Este trabalho foi submetido e aprovado pelo Comitê de Ética em Pesquisa (protocolo 506.070). Não foi necessário consentimento informado especial diferente daquele aplicado a todos os pacientes cirúrgicos. Foi realizado no Hospital Israelita Albert Einstein, ativamente questionando pacientes operados entre 2004 e 2013.

### Análise estatística

Para comparar a evolução dos sintomas de DRGE, a GV e a GV + TB foram analisadas juntas e comparadas a GVAR e GVAR + TB juntos. Pacientes que pioraram ou ficaram inalterados foram agrupados e comparados àqueles que melhoraram e ficaram assintomáticos. A comparação foi obtida pelo teste χ^2 ^de Pearson para proporções. O programa utilizado foi R Core Team (Vienna, 2013).

Para comparar a média de perda de peso em cada período separadamente, 3 e 6 meses, 1, 2, 3, 4 e 5 anos, foram utilizados médias e intervalos de confiança de 95%.

## RESULTADOS

Os pacientes para GVAR (38 indivíduos) e GVAR + TB (50 indivíduos) foram operados por todos os quatro autores, do mesmo grupo, de 2004 a 2012. O seguimento foi de 3 a 89 meses (média 22 meses). Dentre os pacientes, 56 eram mulheres e 32 homens. IMC pré-operatório médio para GVAR foi de 38,7kg/m^2^ e para GVAR + TB de 41,8kg/m^2^, enquanto os 360 pacientes com GV tinham IMC médio de 39,2kg/m^2^ e 42,3kg/m^2^ para 1.140 pacientes submetidos a GVAR + TB.

Para 38 pacientes com GVAR, a redução percentual média de EBMIL foi de 52%±4, 74%±5, 79%±7, 72%±8, 75%±7, 62%±8 e 60%±8, respectivamente, aos 3, 6 meses, 1, 2, 3, 4 e 5 anos, ao passo que para GV apenas (360 pacientes) foi, respectivamente, de 49%±3, 72%±5, 84%±6, 79%±7, 74%±7, 60%±7 e 57%±8.

Para GVAR+BT a redução percentual média de EBMIL foi 50%±5, 76%±5, 92%±7, 93%±7, 84%±7, 83%±7 e 80%±8, respectivamente, aos 3, 6 meses, 1, 2, 3, 4 e 5 anos, enquanto para GV + BT sem procedimentos para tratar ou prevenir DRGE os resultados observados foram 47%±6, 72%±6, 92%±7, 95%±7, 86%±6, 79%±7 e 79%±8 (Figura 2).

**Figura 2:** À esquerda, uma comparação do percentual de perda de excesso de índice de massa corporal entre a gastrectomia vertical usual (colunas azuis) e a gastrectomia vertical antirrefluxo (colunas vermelhas). No gráfico à direita, uma comparação entre o excesso de índice de massa corporal de gastrectomia vertical + derivação biliopancreática parcial (colunas azuis) e gastrectomia vertical antirrefluxo + bipartição do trânsito (colunas vermelhas)

Com intervalo de confiança de 95%, a estatística demonstrou que, em todos os períodos analisados, em termos de perda de peso, GVAR + TB não foi inferior a GV + TB somente

Usando o mesmo método estatístico, comparando apenas GV e GVAR, o tamanho da amostra foi menor (38 pacientes), e em dois períodos (1 e 2 anos no pós-operatório) não foi possível afirmar que GVAR não fosse inferior a GV, mas, em todos os outros períodos (3 meses, 6 meses, 3, 4 e 5 anos), a perda de peso obtida com GVAR não foi inferior a GV somente.

### Evolução dos sintomas de doença do refluxo gastresofágico

Dos 50 pacientes submetidos a GV somente que não tinham sintomas pré-operatórios de DRGE, 19% começaram a apresentar alguns sintomas, especialmente nos primeiros 3 meses após a cirurgia. Necessitaram IBPs (esporádico ou não contínuo 13%; contínuo 6%). Assim, GV pôde induzir sintomas de DRGE naqueles que não os tinham previamente.

Entre os pacientes que tinham sintomas típicos de DRGE (azia ou sensação de refluxo) no pré-operatório, pacientes com GV e GV + BT sem procedimentos para tratar ou prevenir DRGE foram analisados em conjunto.

Em relação às queixas relacionadas à DRGE previamente à cirurgia, 15% pioraram, 38% permaneceram inalterados, 41% melhoraram e apenas 6% referiram que não apresentavam mais sintomas. Desse modo, GV pode tanto melhorar quanto piorar os sintomas. Esse grupo de pacientes já utilizava IBPs antes da cirurgia. Após a cirurgia nesse grupo, apenas 12% não utilizaram mais IBPs; enquanto 88% usaram (19% em uso contínuo e 78% esporádico).

Em relação aos pacientes que fizeram GVAR, foram analisados em conjunto quanto aos sintomas de DRGE (GVAR e GVAR + TB) e todos tinham sintomas prévios à cirurgia. Após GVAR, nenhum paciente relatou piora, 2 (2,3%) permaneceram inalterados, 32 (36,4%) relataram melhora e 54 (61,4%) permaneceram assintomáticos. Para a análise estatística, pior e inalterado foram agrupados, assim como melhor e assintomático, conforme demonstrado na figura 3. Comparando com os pacientes sintomáticos que não fizeram hiatoplastia com cardioplicatura, estes resultados foram significativamente melhores (p<0,001).

**Figura 3:** Comparação da evolução dos sintomas da doença do refluxo gastresofágico entre a gastrectomia vertical usual ou gastrectomia vertical + derivação biliopancreática parcial e a gastrectomia vertical antirrefluxo ou gastrectomia vertical antirrefluxo + derivação biliopancreática parcial (acima). Abaixo, uma análise simplificada, na qual “pior e inalterado” estão juntos, assim como “melhor e assintomático”. Observa-se diferença estatística. Os pacientes que receberam os procedimentos antirrefluxo apresentam significativamente menos sintomas (p<0,001)

Isso também foi demostrado quando o uso de IBPs foi analisado. De 100% de uso de IBPs contínuo ou esporádico nesse grupo, o uso caiu para 36,3% (2,3% contínuo e 34% esporádico); 63,7% não precisaram mais de IBPs (p<0,01).

Disfagia persistente a sólidos ocorreu apenas em um paciente após GVAR, mas não foi realizada reintervenção. As complicações cirúrgicas foram raras: um caso de GVAR com sangramento pós-operatório, que necessitou transfusão mas não reintervenção, e um caso de suboclusão intestinal em GVAR + TB, que tampouco necessitou reintervenção.

A manometria pré-operatória em GVAR e GVAR + TB demonstrou pressão respiratória média anormalmente baixa no EEI, variando de 4 a 15mmHg (8,6±3,2mmHg). Raramente foi feita no pós-operatório, em vista de sua baixa aceitação pelos pacientes assintomáticos. Esses dados não foram utilizados para comparação.

Radiografia gastresofágica alta usando bário oral como contraste (série do trato gastrintestinal superior) foi a mais utilizada como meio de avaliar objetivamente os resultados cirúrgicos. Nenhuma hérnias de hiato (21 de 88 pacientes) foi mais detectada. Cinquenta pacientes foram submetidos a série de trato gastrintestinal superior (Figura 2): 44 exames normais, só observando o GV e a plicatura (Figura 4), e 6 exames demonstraram algum refluxo leve; 5 exames também apresentaram algumas ondas esofágicas terciárias. A manobra de Valsava e a posição de Trendelenburg são parte do protocolo em séries de trato gastrintestinal superior e alguns pacientes, mesmo nessa situação adversa, não apresentam refluxo ao exame (Figura 4; observe que na posição de Trendelenburg e durante a manobra de Valsava, os estômagos parecem maiores do que na posição ortostática usual e o meio de contraste está principalmente na porção superior do estômago). Os 38 pacientes sem exame pós-operatório objetivo estavam completamente assintomáticos.

**Figura 4:** Radiografias do trato gastrintestinal alto com bário após gastrectomia vertical antirrefluxo. As três imagens no alto mostram os pacientes em posição ortostática. Nas três inferiores, os pacientes estão em posição de Trendelenburg (nota-se que o contraste se acumula no alto do estômago) e em manobra de Valsava (o estômago está comprimido contra o diafragma). Não se observa refluxo esofágico nesses casos, mesmo em condições adversas. A imagem inferior, à direita, mostra gastrectomia vertical antirrefluxo + derivação biliopancreática parcial: atenção para a anastomose gastroileal laterolateral no antro

## DISCUSSÃO

GV está se tornando cada vez mais frequente e importante como procedimento cirúrgico digestivo. Pesquisa internacional detalhada sobre procedimentos bariátricos mostrou que, entre 2008 e 2011, GV foi a única técnica com aumento em números absolutos, e não por pouco: foi de 18.098 para 94.689, ou seja, um aumento de 523%, enquanto todos os outros procedimentos diminuíram em números absolutos.^(13)^

A GV provou-se segura e eficaz, com perda de peso significativa e melhora de um número expressivo de alterações metabólicas, incluindo o diabete.

Entretanto, a GV tem também suas falhas. O ponto mais fraco desse procedimento é o fato de que, em alguns pacientes, pode causar ou piorar a DRGE. A segunda maior falha é que pode não ser suficiente para todos os pacientes como tratamento de obesidade severa e síndrome metabólica grave. Alguns pacientes podem necessitar de mais ondutas (intervenções que envolvam também o intestino).

Em relação aos sintomas de DRGE, alguns pacientes melhoram dos sintomas após GV, enquanto outros pioram, conforme mencionado na literatura^(14-16,21)^ e aqui confirmado. As implicações de GV no DRGE são múltiplas e muito complexas.^(21)^

Há argumentos a favor de melhora da DRGE após GV. De fato, os pacientes perdem peso, o que ajuda a melhorar a DRGE. No entanto, há outras explicações teóricas para essa melhora, que podem ocorrer antes da perda de peso.^(16)^ A GV remove a maioria das células oxínticas, o que provavelmente reduz, de forma significante, a produção de ácido (embora possa ser óbvio, nunca foi comprovado de modo apropriado). Além disso, GV teoricamente reduz a tensão nas paredes gástricas abaixo do cárdia na mesma pressão interna, já que reduz o raio do fundo gástrico. A lei de LaPlace foi invocada para essa explicação:^(16,20)^ quanto maior o raio do vaso, maior a tensão da parede necessária para aguentar uma determinada pressão interna. A tensão nas paredes do cárdia é a força opositora à ação do EEI. Alguns autores documentaram bem uma melhora na pressão do EEI^(16)^ anterior à perda de peso que pode refletir essa hipótese teórica.

No entanto, na direção oposta, GV eleva a pressão intragástrica, à medida em que reduz a complacência gástrica.^(22)^ Se a pressão intragástrica está muito aumentada, mesmo com um raio pequeno, a tensão da parede pode aumentar e o refluxo pode aparecer ou piorar.

Na GV, o esvaziamento gástrico mais rápido é demonstrado no início das refeições,^(23)^ o que é positivo na prevenção e no tratamento de RGE, mas, em um determinado ponto, o esvaziamento gástrico é reduzido (principalmente pela ação de hormônios intestinais, como o GLP-1),^(24)^ e a pressão intragástrica aumenta. É um cenário complexo de prós e contras.

Há outro ponto crucial na discussão envolvendo GV e DRGE. Desde 1980, houve uma revolução no entendimento de DRGE. Antes de 1980, o refluxo era considerado o resultado de um EEI fraco. Dent et al. descobriram que a maior parte dos eventos de RGE, tanto em indivíduos normais quanto em pacientes com DRGE, era produto de breves relaxamentos do EEI. Estes foram mais tarde denominados relaxamentos transitórios do EEI (RTEEI).^(25,26)^

RTEEI são o mecanismo mais importante para a ocorrência de RGE. RTEEI são relaxamentos que não são desencadeados proximalmente como aqueles que seguem a deglutição. RTEEI são desencadeados distalmente por distensão gástrica, duram mais tempo e ocorrem junto do relaxamento da crura diafragmática. Ocorrem em pessoas normais e são responsáveis por eructação, um evento fisiológico.^(26)^ Os pacientes magros com DRGE apresentam RTEEI mais frequentes, assim como os pacientes com obesidade mórbida.^(27)^

RTEEI ocorrem periodicamente e são desencadeados por eventos mioelétricos de controle neural gerados no fundo gástrico e na cárdia.^(26)^ Portanto, é possível que a ressecção do fundo gástrico remova as fontes de RTEEI.

Comparando pacientes submetidos a fundoplicaturas de Nissen tradicionais, RTEEI (desencadeados por distensão do fundo gástrico por ar) foram significativamente mais elevados em pacientes que tinham os vasos curtos intactos do que naqueles que os tinham seccionados.^(28)^ Isso sugere que simplesmente seccionar os vasos (e conexões neurais) causa diminuição nos RTEEI. É razoável aceitar que uma GV pode causar diminuição importante nos RTEEI, mas isso ainda não foi demonstrado em ambos, GV usual ou GVAR.

Se parte do fundo é mantida após a GV, sua distensão pode começar a desencadear RTEEI frequentes. De fato, no grupo com GV, observamos dois pacientes que desenvolveram eructações extremamente frequentes logo após a GV, sintoma que levou meses para desaparecer. Isso foi provavelmente causado por RTEEI indesejados mais frequentes.

Por esse raciocínio, a melhor GV seria aquela na qual a linha de grampos estivesse bem próxima da junção gastresofágica. Isso é exatamente o que Petersen et al.^(16)^ observaram e sugeriram, no mesmo artigo em que afirmaram que GV aumenta a pressão do EEI.

No entanto, chegar próximo à junção gastresofágica pode ser perigoso, não apenas pelo risco de fístula, mas também em função do perigo de lesar as fibras oblíquas do EEI. Braghetto et al.^(15)^ demonstraram claramente que é possível lesar essas fibras reduzindo a pressão basal do EEI e causando RGE.

Nesse cenário complexo, obter a melhor GV não é fácil, já que não é aconselhável grampear nem muito próximo à junção gastresofágica, nem muito longe.

Após décadas de cirurgia antirrefluxo com sucesso, temos dados sobre o efeito da fundoplicatura nos RTEEI. Tanto fundoplicaturas parciais^(29)^ quanto completas (Nissen)^(30)^ reduziram a ocorrência de RTEEI. A cirurgia antirrefluxo é um tratamento eficaz contra DRGE. Previne RGE de forma eficiente e, às vezes, fundoplicatura completa também impede eructação.^(30)^

Frequentemente, cirurgiões associam a eficácia da fundoplicatura ao tamanho do envelope em torno do esôfago, ou seja, um envelope de 360° seria superior a um de 270°, que, por sua vez, seria melhor do que um de 180**°**. Não há suporte objetivo para essa ideia. Na verdade, em uma meta-análise abrangente e recente,^(31)^ foi demonstrado o oposto, e a fundoplicatura de 180° apresentou resultados globais superiores.

GVAR, como proposto aqui, remove coxins adiposos em torno do esôfago, corrige hérnias de hiato, protege a junção gastresofágica de ser seccionada; portanto protege as fibras oblíquas e mantém bastante o ângulo de His. A plicatura ainda visa manter o pequeno diâmetro do “novo tubo gástrico”, gerando menos tensão na parede (menos distensão como consequência) do cárdia remanescente. Cria, ainda, tração ao nível do EEI.

Objetivamente, GVAR causou uma redução expressiva nos sintomas de DRGE e na necessidade de IBPs quando comparado a GV (com ou sem BT). Embora não tenhamos dados suficientes em relação às mudanças na pressão do EEI nem na frequência de RTEEI, GVAR foi eficaz na redução dos sintomas de DRGE. Obviamente, saber exatamente como essa pequena plicatura afeta a pressão do EEI e a frequência de RTEEI enriqueceria essa discussão. Estudos científicos são necessários, mas, por outro lado, há suporte científico para poupar pacientes assintomáticos de exames invasivos, uma vez que a avaliação de sintomas no seguimento também é adequada após fundoplicaturas, e testes fisiológicos de rotina não são necessários para pacientes assintomáticos^(32)^ (especialmente porque eles não os querem repetir).

GVAR + BT foi aplicada aos pacientes mais pesados, aqueles com síndrome metabólica mais intensa ou aqueles com limitações severas a exercício, nos quais a perda de peso ideal é menos provável e mais importante. A perda de peso (em termos de EBMIL%) foi melhor do que GVAR apenas, e não inferior a GV + TB sem procedimentos antirrefluxo, em todos os procedimentos e em todos os períodos examinados, demonstrando que a cardioplicatura não prejudica a perda de peso. BT cria uma anastomose gastroileal maior, que pode evitar a elevação da pressão intragástrica depois de GV e, como consequência, os resultados relativos a DRGE podem ser até melhores, mas isso não foi ainda comprovado.

Tai et al.^(14)^ bem demonstraram que, após GV e a perda de peso esperada, hérnias de hiato frequentemente apareciam. É razoável, pois geralmente há muita gordura em torno da transição gastresofágica que o emagrecimento pode diminuir, deixando o hiato alargado. GVAR remove os coxins de gordura e corrige eventuais hérnias de hiato, garantindo que a junção gastresofágica esteja no abdômen e, por fechar a crura, é possível que atenue a intensidade do relaxamento diafragmático, que ocorre simultaneamente aos RTEEI, como também imagina-se que aconteça na hiatoplastia das fundoplicaturas tradicionais.

Fixando o estômago em posição após GV,^(20)^ a torção do estômago pode ser prevenida. Isso pode contribuir para um menor gradiente de pressão para obter esvaziamento gástrico. Esse fato, no entanto, embora intuitivo, nunca foi comprovado objetivamente. Facilitar o esvaziamento gástrico é reconhecido como parte do tratamento para DRGE.

Este artigo originalmente descreveu que alguns procedimentos usuais e simples no tratamento cirúrgico da DRGE podem ser aplicados à GV. O vídeo anexo os demonstra (http://learning.einstein.br/ao2885). A observação dos resultados nesse grupo é estimulante. No entanto, o estudo é retrospectivo e não randomizado, e medidas precisas e objetivas não foram demonstradas. Isso demanda estudos adicionais.

## CONCLUSÃO

O acréscimo de procedimentos antirrefluxo (hiatoplastia, remoção dos coxins de gordura, fixação do estômago na posição anatômica e cardioplicatura) à gastrectomia vertical tradicional, neste grupo, não aumentou as morbidades e nem piorou a perda de peso, mas reduziu significativamente a ocorrência de sintomas de refluxo gastresofágico, assim como o uso de inibidores da bomba de prótons. Estudos prospectivos randomizados adicionais são necessários para avaliar essas modificações técnicas.
